# Oral cancer knowledge, attitudes, and practices among senior dental students in Yemen: a multi-institution study

**DOI:** 10.1186/s12903-023-03149-x

**Published:** 2023-06-30

**Authors:** Anas Shamala, Esam Halboub, Sadeq Ali Al-Maweri, Hesham Al-Sharani, Mona Al-Hadi, Raheq Ali, Hajer Laradhi, Heba Murshed, Marwan Mansoor Mohammed, Kamran Ali

**Affiliations:** 1 Department of Preventive and Biomedical Science, College of Dentistry, University of Science & Technology, Sanaa, Yemen; 2 Department of Maxillofacial Surgery and Diagnostic Sciences, College of Dentistry, Jazan University, Jazan, Saudi Arabia; 3 College of dental medicine, QU health, Qatar University, Doha, Qatar; 4 Faculty of Dentistry, Department of maxillofacial surgery, Ibb University, Ibb, Yemen; 5 Dental Medicine Postgraduate Program, Faculty of Dental Medicine, Universitas Airlangga, Surabaya, Indonesia; 6 Internship program, College of Dentistry, University of Science & Technology, Sanaa, Yemen; 7 Department of Oral and Craniofacial Health Sciences, College of Dental Medicine, University of Sharjah, Sharjah, United Arab Emirates

**Keywords:** Oral cancer, Knowledge, practice, Dental students, Yemen

## Abstract

**Background:**

The “Knowledge, attitude, and practice (KAP)” approach is crucial in health system. Appreciating the current KAP status will reveal the extent of the efficiency of applied health strategies, and subsequently help to determine the appropriate health policy to be employed for improving the health indicators of a given disease/condition, including Oral Cancer (OC). This large-scale cross-sectional study aimed to assess knowledge, attitude, and practice on OC among senior dental students in Yemen.

**Methods:**

A pre-validated online questionnaire was used for data collection. It consisted of a series of close-ended questions on knowledge, attitudes and practices related to OC. Yemeni dental students in clinical levels (4th and 5th years) from nine dental schools based in four major cities were invited to complete the survey. The SPSS Version 28.0 was used for data analysis. Differences by different grouping factors were assessed by Chi-squared and Mann Whitney-U tests, as appropriate.

**Results:**

A total of 927 students completed the questionnaire: a response rate of 43%. While the majority identified smoking (93.8%), and smokeless tobacco (92.1%) as potential risk factors of OC, only 76.2% recognized sun exposure as a risk factor for lip cancer and only 50% were aware of old age as a risk factor of OC. Regarding clinical signs of OC, 84.1% reported that OC can present as non-healing ulcer, but only two thirds of the participants recognized that OC can present as a white and/or red lesion. With respect to practices, although 92.1% reported asking their patients regarding oral habits, only 78% reported they regularly carry out a soft tissue examination. Only 54.5% of the participants considered themselves to be well-trained to provide smoking cessation advice, and 21% were confident regarding their knowledge on OC. The 5th year students showed significantly better knowledge and practices than the 4th year students did (p < 0.01).

**Conclusion:**

The study suggests significant gaps in knowledge, attitudes, and practices of senior dental students in Yemen regarding OC. The findings also underscore the urgent need to improve OC teaching and training of undergraduate dental students, and to provide periodic well-structured continuing professional development activities for dental professionals.

**Supplementary Information:**

The online version contains supplementary material available at 10.1186/s12903-023-03149-x.

## Introduction

Oral cancer (OC) is a significant global public health problem, with an estimated 377,713 new cases and 177,757 associated deaths in 2020 [1]. Unfortunately, the burden of OC largely affects underdeveloped and developing countries [1]. Oral squamous cell carcinoma (OSCC) is the most common type of OC accounting for over 90% cases. The etiopathogenesis of OC is quite complex, and multiple factors - either individually or synergistically - are involved. Indeed, around 85% of OC cases are preventable as they are attributed to modifiable risk factors including smoking, smokeless tobacco, betel nut, and alcohol, and sun exposure in context of lip and skin cancer [2, 3]. Nevertheless, although the large fraction of OC is preventable as indicated above, along with the increasingly huge advancement in all medical aspects [4], the incidence of OC still on the rise especially among young age groups, and the mortality rates have not declined [2, 5].

Like many other developing countries, cancer remains a major public health issue in Yemen. Although there is no accurate data on the incidence rate of OC in Yemen (due to lack of national cancer registries), data from tertiary oncology centers in Yemen have showed high relative frequencies of OC, and unfortunately most cases were diagnosed in advanced stages [6–8]. Additionally, a relatively recent systematic review on OC epidemiology in the Arab world, revealed that Yemenis have one of the highest incidence rates in the region, especially among young patients < 45 year old [9]. Such a grave scenario may be attributed to a multitude of factors, including the low socioeconomic status, the limited availability of specialist services, lack of public awareness, and the high prevalence of OC risk factors like smoking, smokeless tobacco (locally known as Shammah), and water pipe smoking [10–15]. The ongoing nine-year civil war has also had a negative impact on the economy and health services. The socioeconomic and humanitarian conditions deteriorated markedly, resulting in devastating consequences for the local population and contributed to indulgence of the youth in smoking, shammah use and khat chewing (a deep-rooted habit in the country). Along with that, the almost total collapse in the healthcare system contributed to delay in early diagnosis and treatment of OC [16].

Undoubtedly, early detection of OC is critical for achieving favorable treatment outcomes and higher survival rates [4]. Dentists and allied dental care professional play a pivotal role in the fight against OC and contribute to prevention, early detection, and prompt referral of suspected OC to relevant specialists. However, previous studies suggest gaps in the knowledge and confidence of healthcare providers including dental professionals to detect and refer OC [17–23]. Previous studies on dental students and graduates have also highlighted similar deficiencies in recognition and timely referral to the specialists [18–21, 24–27]. The common factors underlying lack of knowledge and clinical detection of OC amongst dentists and dental students relate to insufficient training and exposure to suspected OC. Data regarding the knowledge, attitudes, and practices on OC among Yemeni dentists and dental students are limited. Apart from one single-institute study on Yemeni dental students [28], which revealed inadequate knowledge and practices among the students, there is limited published literature in this context. Hence, the present study aimed to investigate the knowledge, attitudes and practices of clinical dental students in Yemen regarding OC prevention and early detection.

## Methods

### Ethics approval

The Ethics Committee, Faculty of Dentistry, University of Science and Technology, Sana`a, Yemen, approved the study protocol (No: EAC/UST230). Participation was voluntary, informed consents were obtained from all participants, and all data were processed anonymously.

### Settings

The study was conducted in five dental colleges in four densely-populated cities (Sana’a University and University of Science and Technology, Sana’a city; Ibb University, Ibb city; Aden University, Aden city; and Taiz University, Taiz city). Data collection was done from May to November 2022.

### Study design

Cross-sectional, questionnaire-based study.

### Sampling technique

Convenience sampling was used.

### Participants

The study targeted Yemeni dental students in clinical levels (4th and 5th year undergraduate dental students) in the five dental colleges indicated above.

### Data Collection

A web-based questionnaire was used in the present study using a pre-validated questionnaire, adopted from previous studies [28, 29]. The questionnaire was prefaced with an introductory paragraph to clarify the objective of the study, assure anonymous and voluntary participation, and confirm that the responses will be confidential, and accessible only by the authors. The questionnaire consisted of 38 items divided into 4 parts (Appendix). The first part of questionnaire was about the characteristics and demographic data: Gender, age, university, and whether it is public or private, smoking, academic year (4th versus 5th levels), and city. The second part included 22 questions addressed the knowledge about the epidemiological and most common clinical characteristics of OC, and its risk factors. Except for the question about “the most common intraoral site of squamous cell carcinoma”, the responses to the remaining questions were either “Yes”, “No”, or “I don’t Know”. The third part comprised four questions on the clinical examination and diagnostic steps practiced by the respondents with “Yes”, No”, and “Not sure” as responses. The last part of questionnaire comprised six questions addressing the attitudes of the participants toward OC; responses varied as per the questions (see the tables). The questionnaire was prepared as Google Form, and then distributed to the targeted sample (year 4, 5 students) via different means of social media (WhatsApp, Facebook, and Telegram). Reminders were sent three times, with two-week intervals. Responses to the questionnaire were sensitive to the Internet Protocol (IP) Address assuring no duplicated responses. The research was self-funded and there are no conflicts of interest to be reported.

### Statistical analysis

The data were obtained as excel file and exported for analysis into SPSS (IBM Corp. Released 2021. IBM SPSS Statistics for Windows, Version 28.0. Armonk, NY: IBM Corp). Data were presented as frequencies and percentages. Differences in responses to the items of the questionnaire were assessed using Chi-squared test. In order to provide an overall score for the items which assessed knowledge and practice (where there were correct answers), each correct response was given a score of 1 while incorrect responses received a score of zero. yielding a maximum score of 22 for knowledge-based items and a maximum score of 4 for practice-related items. Kolmogorov-Smirnov test showed that scores for both domains were abnormally distributed. Therefore, the differences in these scores by the different demographic factors were assessed using Mann Whitney-U test. The level of significance was set at P < 0.05.

## Results

A total of 927 undergraduate dental students in clinical levels (4th and 5th year) from four main cities in Yemen participated in the present study, with an overall response rate of 43% (response rate in different cities: Sana’a, 47%; Aden, 44%; Ibb, 41%; and Taiz, 40%). Number of participants and the response rate from each city are presented in Supplementary Table 1. Of these, 573 (62%) were females and 351 (38%) were males. The mean age of the participants was 23.13 ± 1.42 years. Around 67% of the respondents were from public universities, while the remaining (33%) were from private universities. The demographic data of the study participants are summarized in Table 1.

**Table 1:** Demographic information of the study sample

Variable	Subgroups	n (%)
Gender (N = 924)	Males	351 (38)
	Females	573 (62)
Study levels (N = 927)	4th	525 (56.6)
	5th	402 (43.4)
Smoking (N = 919)	Yes	132 (14.4)
	No	787 (85.6)
University (N = 927)	Public	621 (67)
	Private	306 (33)
City (N = 927)	Sana’a	446 (48.1)
	Aden	143 (15.4)
	Taiz	224 (24.2)
	Ibb	114 (12.3)
Age (N = 823); Mean ± SD*		23.13 ± 1.42

*SD: standard deviation

Most of the participants (86.5%) recognized that OSCC is the most frequent type of OC. However, only 61.6% of the students knew that the tongue and floor of the mouth are the most common sites of occurrence, and only 59% were aware that OC is typically discovered in the late stage (Table 1).

Regarding knowledge on the risk factors of OC, most of the students correctly reported smoking (93.8%), smokeless tobacco (92.1%), alcohol intake (82%), immunosuppression (76.5%), sun exposure for lip cancer (76.2%), and chronic trauma (72.8%) as OC risk factors. However, fewer students reported viral factors (65.9%), ill-fitting dentures (64.1%) and old age (51.6%) as risk factors (Table 2).

**Table 2:** Knowledge on oral cancer, and its risk factors and clinical signs among dental students by the year of study

Questions	Responses	Study level		Total	p value
		4th	5th		
***Demographics***					
Squamous cell carcinoma is the most common form of oral cancer (N = 904)	No	26 (5.1)	14 (3.5)	40 (4.4)	**< 0.001**
	Yes	414 (81.5)	368 (92.9)	782 (86.5)	
	I don’t know	68 (13.4)	14 (3.5)	82 (9.1)	
The most common site of Squamous cell carcinoma (N = 915)	Tongue and FOM*	275 (53.3)	289 (72.4)	564 (61.6)	**< 0.001**
	Buccal mucosa	149 (28.9)	69 (17.3)	218 (23.8)	
	Gingiva	45 (8.7)	14 (3.5)	59 (6.4)	
	Lips	47 (9.1)	27 (6.8)	74 (8.1)	
Squamous cell carcinomas mostly diagnosed at advanced stage (N = 857)	No	135 (27.8)	95 (25.6)	230 (26.8)	**0.003**
	Yes	267 (54.9)	239 (64.4)	506 (59)	
	I don’t know	84 (17.3)	37 (10)	121 (14.1)	
***Risk factors***					
Qat chewing (N = 916)	No	164 (31.5)	136 (34.3)	300 (32.8)	0.484
	Yes	313 (60.2)	234 (59.1)	547 (59.7)	
	I don’t know	43 (8.3)	26 (6.6)	69 (7.5)	
Smoking (N = 918)	No	34 (6.5)	16 (4)	50 (5.4)	0.188
	Yes	438 (92.5)	378 (95.5)	861 (93.8)	
	I don’t know	5 (1)	2 (0.5)	7 (0.8)	
Smokeless tobacco use (N = 916)	No	35 (6.8)	17 (4.3)	52 95.7)	0.115
	Yes	468 (90.5)	376 (94.2)	844 (92.1)	
	I don’t know	4 (2.7)	6 (1.5)	10 (2.2)	
Alcohol consumption (N = 915)	No	70 (13.5)	33 (8.3)	103 (11.3)	**0.021**
	Yes	409 (79)	341 (85.9)	750 (82)	
	I don’t know	39 (7.5)	23 (5.8)	62 (6.8)	
Viral factors (N = 911)	No	112 (21.7)	82 (20.8)	194 (21.3)	**0.030**
	Yes	326 (63.1)	274 (69.5)	600 (65.9)	
	I don’t know	79 (15.3)	38 (9.8)	117 (12.8)	
Sun exposure for lip cancer (N = 911)	No	91 (17.5)	50 (12.8)	141 (15.5)	**< 0.001**
	Yes	370 (71.2)	324 (82.9)	694 (76.2)	
	I don’t know	59 (11.3)	17 (4.2)	76 (8.3)	
Immunosuppression (N = 903)	No	70 (13.7)	49 (12.5)	119 (13.2)	0.811
	Yes	387 (75.7)	304 (77.6)	691 (76.5)	
	I don’t know	54 (10.6)	39 (9.9)	93 (10.3)	
Chronic trauma (N = 913)	No	133 (25.7)	43 (10.9)	176 (19.3)	**< 0.001**
	Yes	333 (64.3)	332 (84.1)	665 (72.8)	
	I don’t know	52 (10)	20 (5.1)	72 (7.9)	
Older age (N = 909)	No	219 (42.5)	100 (25.4)	319 (35.1)	**< 0.001**
	Yes	219 (42.5)	260 (63.5)	469 (51.6)	
	I don’t know	77 (15)	44 (11.2)	121 (13.3)	
Low consumption of fruits and vegetables (N = 900)	No	272 (53.4)	191 (48.8)	463 (51.4)	**0.031**
	Yes	139 (27.3)	138 (35.3)	277 (30.8)	
	I don’t know	98 (19.3)	52 (15.9)	160 (17.8)	
Family history of cancer (N = 914)	No	122 (23.5)	91 (23.1)	213 (23.3)	0.146
	Yes	358 (68.8)	285 (72.2)	643 (70.4)	
	I don’t know	40 (7.7)	18 (4.6)	58 (6.3)	
Poor oral hygiene (N = 906)	No	156 (30.4)	127 (32.3)	283 (31.2)	0.598
	Yes	309 (60.2)	236 (60.1)	545 (60.2)	
	I don’t know	48 (9.4)	30 (7.6)	78 (8.6)	
Poorly fitting denture (N = 916)	No	155 (29.8)	72 (18.2)	227 (24.8)	**< 0.001**
	Yes	291 (55.9)	296 (74.9)	587 (64.1)	
	I don’t know	75 (14.4)	27 (6.8)	102 (11.1)	
***Clinical signs***					
Non-healing ulcer (N = 914)	No	50 (9.6)	25 (6.3)	75 (8.2)	**< 0.001**
	Yes	413 (79.4)	356 (90.4)	769 (84.1)	
	I don’t know	57 (11)	13 (3.3)	70 (7.7)	
Red lesion (N = 903)	No	114 (22.3)	86 (22)	200 (22.1)	0.074
	Yes	320 (62.5)	265 (67.8)	585 (64.8)	
	I don’t know	78 (15.2)	40 (10.2)	118 (13.1)	
White lesion (N = 902)	No	95 (18.7)	70 (17.8)	165 (18.3)	**< 0.001**
	Yes	327 (64.2)	294 (74.8)	621 (68.8)	
	I don’t know	87 (17.1)	29 (7.4)	116 (12.9)	
Speckled (red and white) lesion (N = 897)	No	61 (12)	29 (7.5)	90 (10)	**< 0.001**
	Yes	358 (70.5)	324 (83.3)	682 (76)	
	I don’t know	89 (17.5)	36 (9.3)	125 (13.9)	
Lump (N = 906)	No	48 (9.4)	50 (12.7)	98 (10.8)	0.110
	Yes	420 (81.9)	319 (81.2)	739 (81.6)	
	I don’t know	45 (8.8)	24 (6.1)	69 (7.6)	
Bleeding (N = 907)	No	191 (37.1)	133 (33.9)	324 (35.7)	**0.007**
	Yes	212 (41.2)	199 (50.8)	411 (45.3)	
	I don’t know	112 (21.7)	60 (15.3)	172 (19)	

*FOM: floor of the mouth

The participants showed a good knowledge about most of the clinical signs associated with OC: presentation as a non-healing ulcer (84.1%), a red lesion (64.8%), a white lesion (68.8%), a speckled lesion (76%) or a lump (81.6%). However, only 45.3% reported bleeding as a possible clinical sign of OC. Generally, 5th year students showed better knowledge of OC compared to 4th year students; there were significant differences between the two groups in most of the items related to knowledge (p < 0.05; Table 2).

Regarding the practices and attitudes of dental students towards OC prevention and early detection, most of the students (92.1%) reported asking their patients about the use of tobacco, and 83.1% reported advising patients to quit tobacco. Around 79% of the participants reported experience of clinical examination of oral soft tissues, but less than half of the participants reported that they had the opportunity to examine patients with a suspicious oral lesion. Moreover, only 54.5% of the participants reported that they had received enough training to provide smoking cessation advice; 36.7% reported that they have sufficient knowledge on prevention and detection of OC; and 21% reported being well-informed about OC. Around 35.2% reported that the university training was adequate for OC examination, while 88.3% of students expressed the need for more information on OC-related topics (Table 3).

**Table 3:** Practice and attitude of dental students regarding oral cancer prevention, diagnosis, and treatment by the year of study

Questions	Responses	Study level		Total	p value
		4th	5th		
***Practices***					
Do you routinely ask patients if they use tobacco? (N = 915)	No	42 (8.1)	30 (7.6)	72 (7.9)	0.805
	Yes	477 (91.9)	366 (92.4)	843 (92.1)	
Do you advise your patients to quite tobacco? (N = 912)	No	107 (20.7)	47 (11.9)	154 (16.9)	**< 0.001**
	Yes	409 (79.3)	349 (88.1)	758 (83.1)	
Do you examine patient’s oral mucosa routinely? (N = 907)	No	110 (21.4)	81 (20.6)	191 (21.1)	0.805
	Yes	403 (78.6)	313 (79.4)	716 (78.9)	
Have you had the opportunity to examine patients with a suspicious oral lesion? (N = 880)	No	248 (50.1)	161 (41.8)	409 (46.5)	**0.002**
	Yes	207 (41.8)	205 (53.2)	412 (46.8)	
	Not sure	40 (8.1)	19 (4.9)	59 (6.7)	
***Attitudes***					
Do you feel adequately trained to provide tobacco cessation advice? (N = 901)	No	190 (37.5)	123 931.2)	313 (34.7)	**0.008**
	Yes	254 (50.1)	237 (60.2)	491 (54.5)	
	Not sure	63 (12.4)	34 (8.6)	97 (10.8)	
As regards to the clinical appearance of oral cancer, how knowledgeable do you feel? (N = 879)	Poorly informed	355 (71.1)	245 (64.5)	600 (68.3)	0.065
	Well informed	92 (18.4)	94 (24.7)	186 (21.2)	
	Very well informed	52 (10.4)	41 (10.8)	93 (10.6)	
Do you consider that the university provided adequate training on oral cancer examination? (N = 892)	No	296 (58.8)	218 (56)	514 (57.6)	**0.001**
	Yes	159 (31.9)	155 (39.8)	314 (35.2)	
	Not sure	48 (9.5)	16 (4.1)	64 (7.2)	
Do you feel that you have sufficient knowledge concerning prevention and detection of oral cancer? (N = 897)	No	320 (63.2)	203 (51.9)	523 (58.3)	**0.002**
	Yes	161 (31.8)	168 (43)	329 (36.7)	
	Not sure	25 (4.9)	20 (5.1)	45 (5)	
Would you like more information or teaching on oral cancer? (N = 897)	No	48 (9.5)	37 (9.5)	85 (9.5)	0.463
	Yes	444 (87.7)	348 (89)	792 (88.3)	
	Not sure	14 (2.8)	6 (1.5)	20 (2.2)	

The mean and median scores of OC knowledge and practice by domain are presented in Table 4. Students in the 5th year showed significantly higher knowledge and practice scores than students in 4th year did (P < 0.001). Additionally, students in public universities showed slightly better knowledge scores than those from the private universities did (p = 0.021). However, no significant differences were found with respect to the practice scores. Gender of the participants did not show significant differences in the overall knowledge and practice scores (Table 4), yet there were some differences in some individual items (Supplementary Tables 1, 2). Individual items in the questionnaire that had significant differences university-wise are shown in Table S2. Additionally, KAP items with unsatisfactory responses are presented in supplementary Fig. 1.

**Table 4:** Mean and median scores of knowledge and practice by demographic factors

Variable	Categories (N/N)	Knowledge score		P value	Practice score		P value
		Median (IQR)	Mean ± SD		Median (IQR)	Mean ± SD	
Stage of study	Year 4(415/489)	15 (12–17)	14.65 ± 3.45	**< 0.001**	3 (2 − 4)	2.91 ± 0.93	**< 0.001**
	Year 5 (342/382)	17 (14–19)	16.44 ± 3.54		3 (3–4)	3.14 ± 0.88	
Smoking	No (657/744)	15 (13–18)	15.48 ± 3.64	0.555	3 (3–4)	3.03 ± 0.9	0.199
	Yes (98/121)	15 (13–17)	15.28 ± 3.33		3 (2–4)	2.88 ± 1.02	
Gender	Males (297/333)	15 (13–18)	15.36 ± 3.84	0.778	3 (2–4)	2.92 ± 1	0.117
	Females (458/537)	15 (13–18)	15.51 ± 3.44		3 (3–4)	3.06 ± 0.86	
University	Private (199/274)	15 (13–17)	14.94 ± 3.34	**0.021**	3 (2–4)	2.95 ± 0.88	0.063
	Public (558/598)	16 (13–18)	15.64 ± 3.68		3 (3–4)	3.04 ± 0.93	

All tests were conducted using Mann-Whitney test. N/N: number of responses in knowledge and practice, respectively; IQR: interquartile range; SD: standard deviation

**Fig. 1:**
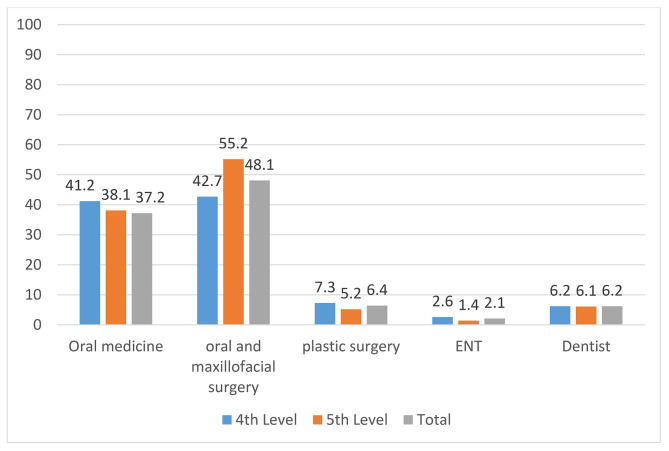
Participants’ referral preference of suspected oral cancer cases (%)

For a patient with a potential diagnosis of OC, the majority of students suggested referral to oral and maxillofacial surgeons (48.1%) and oral medicine specialists (37.2%; Fig. 1).

## Discussion

This is the first large-scale, multi-institution study to evaluate OC knowledge and practices among clinical dental students in Yemen. Given the large sample size from nine Universities located in four major cities, the data may be considered to be representative of all clinical dental students in Yemen. The present findings should be interpreted with caution considering the relative low response rate (43%), which might have introduced a non-response bias, and thus the generalizability of the results is questionable. However, it should be noted that this response rate is reasonable and comparable to other web-based surveys among dental students and professionals worldwide [22, 26, 28, 30]. Overall, the results showed that although the participants had a fair knowledge of OC, significant gaps regarding OC prevention and early detection were identified. While the majority correctly identified tobacco and alcohol as risk factors of OC, only two thirds of the students recognized sun exposure as a risk factor for lip cancer and only 50% reported old age as a risk factor of OC. Low confidence was reported by the participants to provide smoking cessation advice as only 45% thought they are well-trained to provide advice such. Only 21% were satisfied with their knowledge of OC. The results also showed that 5th year students had significantly better knowledge and practices than the 4th year students (P < 0.01 each).

One of the key findings of this study relates to the inadequate knowledge the participants showed about the risk factors and clinical signs of OC. Although the vast majority of the participants identified tobacco and alcohol as the main risk factors, a considerable proportion were not aware that other factors such as age, viral factors, sun exposure are also potential risk factors. These results are consistent with other studies conducted in Saudi Arabia [22], Romania [23], and Turkey [24]. Similarly, the students revealed fair knowledge regarding the clinical signs of OC, a finding similar to those of many previous studies elsewhere [25–27]. Regarding the practices of OC prevention and early detection, the present survey demonstrated a relatively good level of practice among the participants. Nevertheless, some areas were flagged up where their practices need to be improved. The primary prevention of OC is through reducing common risk factors including tobacco habits. In this context, the dental professionals can play pivotal role through educating their patients on the risk factors and advise them to quit deleterious habits such as smoking and other tobacco habits. It is heartening to observe that most of the participants reported asking their patients about their habits and advising them to quit the bad ones. These findings are consistent with many previous studies [22, 23]. The secondary prevention of OC is through screening and early detection using visual examination of oral mucosal. More than three quarters of the participants reported routinely performing clinical oral mucosa examination. This finding is consistent with many previous studies [25–27, 31, 32].

Not surprisingly, the results reveled that 5th year students have significantly higher knowledge and better practices scores than 4th year students. These differences may be attributed primarily to increased clinical exposure and consolidation of knowledge related to OC. This finding is in accordance with that demonstrated among dental students in Palestine [21]. More importantly, the findings underscore the need to further enhance the clinical exposure of dental students and provided regular continuing professional development activities on OC during the undergraduate program and beyond.

Despite students’ interests to actively participate in the early detection and prevention of OC, the low confidence was reported as a barrier against their ability to undertake clinical examination, and provide preventive advice to patients. Previous studies on dental students and new graduates from several regions across the globe highlighted the lack of preparedness to refer cases of suspected OC to specialists.

Recent data from the Global Cancer Observatory shows that the incidence of OC is likely to rise by 40% by 2040 with a corresponding increase in the associated mortality [33]. Therefore, the dental institutions need to adopt robust and multipronged strategies to enhance teaching and training of dental students on OC to ultimately prepare future generations of well-trained dentists. Firstly, the undergraduate dental curricula need to be revisited to ensure a comprehensive coverage and continually updated information on OC clinical manifestation, clinical detection, preventative strategies to address risk factors, and understanding the role of multidisciplinary team (MDT) in the management. Second, active participation of students can be ensured by incorporating student led presentations and workshops including role playing to gain an understanding of referral pathways and preventive advice. Third, it is also important to provide dental students with regular exposure to assessment, screening, diagnosis and follow-up of suspected OC in outpatient settings, MDT clinics, as well as observing patients undergoing cancer surgery and post-operative care in specialist settings. Finally, community outreach programs can be used effectively to raise public awareness about OC and the importance of regular screenings. This will in turn have a positive impact on student learning and help them consolidate their approach to public education and prevention.

In addition, dental education must expose the undergraduate students to the emerging technologies which are currently employed in OC screening. There is growing use of artificial intelligence (AI) in OC screening and detection by frontline healthcare workers. Studies suggest that AI can diagnose cancer with greater accuracy than trained clinicians, a matter that strengthens the argument for its development to provide clinical benefits for the patients [34]. It is only a matter of time that widespread use of AI in cancer diagnosis and treatment planning may become a norm in the fight against cancer [35]. Dental education needs to consider the growing role of technology and train the students in OC using the available AI tools. Eventually, the early detection of OC could be revolutionized by AI, with applications being developed for use by both frontline healthcare workers and the general public.

In our study, lack of training and confidence were reported as the main barriers to performing oral mucosal screening for cancerous and precancerous lesions. Both of them could be managed by enhancing the dental undergraduate curricula and provisioning continuing educational programs and training on OC prevention and early detection. It is worth mentioning that undergraduate dental program in Yemen is a 5- year program, and OC topics is taught in oral medicine, oral pathology and oral surgery in third, fourth and fifth years. According to this study, there might be weakness points in teaching the topics relevant to OC either in the quantity, the strategy or both. Of note, the teaching strategy in Yemen is still following the traditional method with less engagement of students in the learning process. Therefore, an integrated approach to teach OC with great emphasis on clinical training on oral mucosal examination is highly warranted.

The present study has some limitations which need to be acknowledged. Firstly, given the self-reported nature of surveys, the respondents’ answers do not necessarily reflect their real practices and perceptions, along with the fact that the recall bias exists. Secondly, the selection bias is an important potential limitation as the dental students who chose to participate in the survey may be more interested in OC than those who did not participate. Therefore, the results are likely not generalizable to non-respondents. Another limitation is that patients with oral mucosal diseases, like OC, or salivary gland diseases seldom attend dental college seeking diagnosis and treatment. Basically, patients attend therein for dental services. So, the clinical exposure of dental students to these diseases is minimum, or even lacking. Accordingly, their training and practice on these diseases is basically theoretical rather than clinically-oriented. Despite these potential limitations, the present study provides very valuable information on the knowledge and practices of OC among senior dental students in Yemen and may serve as a reference point for future studies.

In conclusion, this study sheds light on notable deficiencies in OC knowledge and practices among senior dental students in Yemen, which may have adverse effects on early detection, referral, and prevention of OC. While the majority of participants acknowledged oral squamous cell carcinoma as the primary type of cancer and identified smoking as a significant risk factor, knowledge gaps were identified regarding the most common intraoral sites for OC, additional risk factors, and providing smoking cessation advice to patients. Furthermore, participants expressed limited clinical experience in examining patients with suspicious oral lesions and expressed a desire for further education and training to enhance their skills in recognizing and referring suspected OC cases. These findings emphasize the urgent need to enhance OC teaching and training for undergraduate dental students and provide structured continuing professional development opportunities for dental professionals. It is recommended that further research be conducted to assess the knowledge and skills of undergraduate dental students, particularly in countries with a high incidence of OC. Additionally, it is important to monitor the impact of increased clinical exposure on students’ abilities to recognize suspicious oral lesions, in order to identify the most effective strategies for improving their capabilities.

## Electronic supplementary material

Below is the link to the electronic supplementary material.

Supplementary Material 1

Supplementary Material 2

## Data Availability

The data supporting the findings of this study are available within the article and its supplementary material.
