# Supplementary material for: Oral cancer knowledge, attitudes, and practices among senior dental students in Yemen: a multi-institution study
Source: BMC Oral Health. 2023 Jun 30;23:435. doi: 10.1186/s12903-023-03149-x (PMC10314541; doi:10.1186/s12903-023-03149-x)
Supplement: Supplementary file 1 — Supplementary Material 1 [file 12903_2023_3149_MOESM1_ESM.docx]

**Supplementary figure 1: KAP items with unsatisfactory answers (%)**
