# Supplementary material for: Oral cancer knowledge, attitudes, and practices among senior dental students in Yemen: a multi-institution study
Source: BMC Oral Health. 2023 Jun 30;23:435. doi: 10.1186/s12903-023-03149-x (PMC10314541; doi:10.1186/s12903-023-03149-x)
Supplement: Supplementary file 2 — Supplementary Material 2 [file 12903_2023_3149_MOESM2_ESM.docx]

**Supplementary Table 1: Number of participants and response rate from each city**

| **City** | **Name of University** | **No. of Response** | | **Total** | **Response rate** |
| --- | --- | --- | --- | --- | --- |
|  |  | **L.4** | **L.5** |  |  |
| **Sana'a** | Sana'a University | 90 | 118 | 208 | 47% |
|  | University of Science & Technology | 166 | 72 | 238 |  |
|  |  | 256 | 190 | 446 |  |
| **Aden** | Aden University | 126 | 17 | 143 | 44% |
| **Ibb** | Ibb University | 41 | 73 | 114 | 41% |
| **Taiz** | Taiz University | 102 | 122 | 224 | 40% |
|  | Total | 525 | 402 | 927 | 43% |

Table S2. Knowledge, practice and attitude on oral cancer among dental students by Gender (Only items which were statistically different were presented)

| Item | Responses | Gender | | P value |
| --- | --- | --- | --- | --- |
|  |  | Males | Females |  |
| ***Demographic*** | | | | |
| The most common site of Squamous cell carcinoma (N = 913) | Tongue and FOM | 163 (56.1) | 369 (64.9) | **0.014** |
|  | Buccal mucosa | 86 (25) | 132 (23.2) |  |
|  | Gingiva | 31 (9) | 28 (4.9) |  |
|  | Lips | 34 (9.9) | 40 (7) |  |
| ***Risk factors*** | | | | |
| Qat chewing (N = 913) | No | 133 (38.4) | 167 (29.5) | **0.004** |
|  | Yes | 196 (56.6) | 348 (61.4) |  |
|  | I don’t know | 17 (4.9) | 52 (9.2) |  |
| Smoking (N = 915) | No | 24 (6.9) | 26 (4.6) | **0.056^*^** |
|  | Yes | 318 (91.6) | 540 (95.1) |  |
|  | I don’t know | 5 (1.4) | 2 (0.4) |  |
| Alcohol consumption (N = 912) | No | 60 (17.4) | 43 (7.9) | **< 0.001** |
|  | Yes | 262 (75.9) | 486 (85.7) |  |
|  | I don’t know | 23 (6.7) | 38 (6.7 |  |
| Viral factors (N = 908) | No | 62 (18) | 132 (23.4) | **0.037** |
|  | Yes | 244 (70.9) | 353 (62.6) |  |
|  | I don’t know | 38 (11) | 79 (14) |  |
| Family history of cancer (N = 911) | No | 115 (33.1) | 96 (17) | **< 0.001** |
|  | Yes | 209 (60.2) | 433 (76.8) |  |
|  | I don’t know | 23 (6.6) | 35 96.2) |  |
| Poor oral hygiene (N = 903) | No | 82 (23.8) | 200 (35.8) | **< 0.001** |
|  | Yes | 246 (71.3) | 297 (53.2) |  |
|  | I don’t know | 17 (4.9) | 61 910.9) |  |
| Poorly fitting denture (N = 913) | No | 83 (23.9) | 144 (25.9) | **0.067** |
|  | Yes | 235 (67.7) | 349 (61.7) |  |
|  | I don’t know | 29 (8.4) | 73 (12.9) |  |
| ***Signs*** | | | | |
| Speckled (white and red) lesion (N = 894) | No | 38 (11.2) | 52 (9.4) | **0.027** |
|  | Yes | 242 (71.4) | 438 (78.9) |  |
|  | I don’t know | 59 (17.4) | 65 (11.7) |  |
| ***Practice*** | | | | |
| Do you advise your patients to quite tobacco? (N = 909) | No | 71 (20.5) | 82 (14.6) | **0.022** |
|  | Yes | 275 (79.5) | 481 (85.4) |  |
| Do you examine patient's oral mucosa routinely? (N = 904) | No | 96 (27.7) | 95 (17) | **< 0.001** |
|  | Yes | 250 (72.3) | 463 (83) |  |
| ***Attitude*** | | | | |
| As regards to the clinical appearance of oral cancer, how knowledgeable do you feel? (N = 877) | Poorly informed | 203 (61.9) | 396 (72.1) | **0.006** |
|  | Well informed | 86 (26.2) | 100 (18.2) |  |
|  | Very well informed | 39 (11.9) | 53 (9.7) |  |
| Do you feel that you have sufficient knowledge concerning prevention and detection of oral cancer? | No | 171 (50.4) | 351 (63.1) | **< 0.001** |
|  | Yes | 147 (43.4) | 181 (32.6) |  |
|  | Not sure |  |  |  |
| When you have graduated, where would you refer a patient if you suspected an oral malignancy? (N = 485) | Oral medicine | 84 (48) | 97 (313) | **0.003** |
|  | Oral and maxillofacial surgery | 68 (38.9) | 165 (53.2) |  |
|  | Plastic surgery | 13 (7.4) | 18 (5.8) |  |
|  | ENT | 2 (1.1) | 8 (2.6) |  |
|  | Dentist | 8 (4.6) | 22 (7.1) |  |

*: close to the statistical significant.

Table S3. Knowledge, practice and attitude on oral cancer among dental students by University (Only items which were statistically different were presented)

| Question | Responses | University | | P value |
| --- | --- | --- | --- | --- |
|  |  | Private | Public |  |
| ***Demographic*** | | | | |
| The most common site of Squamous cell carcinoma (N = 915) | Tongue and FOM | 189 (63.4) | 375 (60.8) | **0.068^*^** |
|  | Buccal mucosa | 70 (23.5) | 148 (24) |  |
|  | Gingiva | 24 (8.1) | 35 (5.7) |  |
|  | Lips | 15 (5) | 59 (9.6) |  |
| Oral cancer lesions are mostly diagnosed in advanced stage (N = 857) | No | 59 (22.6) | 171 (28.7) | **0.039** |
|  | Yes | 171 (65.5) | 335 (56.2) |  |
|  | I don’t know | 31 (11.9) | 90 (15.1) |  |
| ***Risk factors*** | | | | |
| Qat chewing (N = 916) | No | 103 (34.7) | 197 (31.8) | **0.054^*^** |
|  | Yes | 164 (55.2) | 383 (61.9) |  |
|  | I don’t know | 30 (10.1) | 39 (6.3) |  |
| Viral factors (N = 911) | No | 82 (27.8) | 112 (18.2) | **< 0.001** |
|  | Yes | 167 (56.6) | 433 (70.3) |  |
|  | I don’t know | 46 (15.6) | 71 (11.5) |  |
| Immune suppression (N = 903) | No | 52 (17.9) | 67 (10.9) | **< 0.001** |
|  | Yes | 190 (65.3) | 501 (81.9) |  |
|  | I don’t know | 49 (16.8) | 44 (7.2) |  |
| Chronic trauma (N = 913) | No | 80 (26.9) | 96 (15.6) | **< 0.001** |
|  | Yes | 193 (65) | 472 (76.6) |  |
|  | I don’t know | 24 (8.1) | 48 (7.8) |  |
| Old age (N = 909) | No | 147 (50.3) | 172 (27.9) | **< 0.001** |
|  | Yes | 104 (35.6) | 365 (59.2) |  |
|  | I don’t know | 41 (14) | 80 (13) |  |
| Poorly fitting denture (N = 916) | No | 58 (19.4) | 169 (27.4) | **< 0.001** |
|  | Yes | 186 (62.2) | 401 (65) |  |
|  | I don’t know | 55 (18.4) | 47 (7.6) |  |
| ***Signs*** | | | | |
| Non-healing ulcer (N = 914) | No | 36 (12.2) | 39 (6.3) | **0.004** |
|  | Yes | 233 (78.7) | 536 (86.7) |  |
|  | I don’t know | 27 (9.1) | 43 97) |  |
| ***Practice*** | | | | |
| Have you had the opportunity to examine patients with a suspicious oral lesion? (N = 880) | No | 143 (53) | 261 (43.4) | **0.005** |
|  | Yes | 121 (43.4) | 291 (48.4) |  |
|  | Not sure | 10 (3.6) | 49 (8.2) |  |
| ***Attitude*** | | | | |
| Do you feel adequately trained to provide tobacco cessation advice? (N = 901) | No | 104 (36.1) | 209 (341) | **< 0.001** |
|  | Yes | 174 (60.4) | 317 (51.7) |  |
|  | Not sure | 10 (3.5) | 87 (14.2) |  |
| Do you consider that the university provided training on oral cancer examination? (N = 892) | No | 165 (57.1) | 349 (57.9) | **< 0.001** |
|  | Yes | 118 (40.8) | 196 (35.5) |  |
|  | Not sure | 6 (2.1) | 58 (9.6) |  |
| Do you feel that you have sufficient knowledge concerning prevention and detection of oral cancer? (N = 897) | No | 168 (57.6) | 366 (58.5) | **0.004** |
|  | Yes | 117 (40.3) | 212 (34.9) |  |
|  | Not sure | 5 (1.7) | 40 (6.6) |  |

*: close to the statistical significant.
